# Editorial: Glaucoma and Brain: Impact of Neurodegeneration on Visual Abilities and Related Biomarkers

**DOI:** 10.3389/fnagi.2022.919775

**Published:** 2022-05-16

**Authors:** Teresa Rolle, Gemma Caterina Maria Rossi, Paolo Brusini

**Affiliations:** ^1^Eye Clinic, Department of Surgical Sciences, University of Torino, Torino, Italy; ^2^San Matteo Hospital Foundation (IRCCS), Pavia, Italy; ^3^City of Udine Polyclinic, Udine, Italy

**Keywords:** glaucoma, brain, biomarker, neurodegeneration, visual abilities

Glaucoma is a chronic disease characterized by the death of retinal ganglion cells (RGCs) and their axons, which leads to a progressive visual field loss. It is one of the major causes of poor vision worldwide in the elderly; therefore it constitutes a social health emergency whose impact is destined to increase over time: its prevalence is estimated to grow up to 112 million people in 2040 (Quigley and Broman, 2006; Tham et al., 2014; Bourne et al., 2018).

In recent years, there is increased evidence that primary open-angle glaucoma (POAG) is a neurodegenerative disease and similar pathogenetic aspects have been found in other neurodegenerative disorders such as amyotrophic lateral sclerosis, Alzheimer's disease, and Parkinson's disease. In particular, RCGs present apoptosis, a cell death mechanism, that occurs in the Alzheimer's disease. Studies showed that the β-Amyloid deposits and intraneuronal accumulations of hyperphosphorylated tau protein (pTau), specific of Alzheimer's disease, are also involved in the pathogenesis of glaucoma (Wostyn et al., 2010).

Researches carried out in glaucomatous patients highlighted functional and structural changes in the brain. The neurodegenerative process is not limited to the visual pathways but also extends into areas that are not related to the visual system (Gupta and Yücel, 2003, 2007; Gupta et al., 2006; Chang and Goldberg, 2012; Nuzzi et al., 2018). These alterations are correlated with clinical characteristics and severity of the glaucomatous disease. More recent studies proved that glaucoma neurodegeneration is also related to neuroinflammation processes that involve both eye and brain (Rolle et al., 2020).

In clinical practice visual impairment related to glaucomatous disease is quantified with the visual field analysis. As known, this test has several limitations and, above all, it is not able to detect damage at an early stage. Undoubtedly, it would be very interesting to identify other visual abilities that may be altered in glaucomatous patients, closely related to daily life, which may possibly appear in the early stages of the disease.

The purpose of this Research Topic was to publish new researches concerning brain involvement in glaucoma, impairment of other visual abilities and identification of biomarkers for early diagnosis.

Parisi et al. evaluated retinal ganglion cell (RCG) function and the neural conduction along the post-retinal large and small axons and its correlation with retinal nerve fiber layer thickness in open-angle glaucoma patients. In these patients there is a dysfunction involving both post-retinal large and small axons. This abnormal post-retinal neural conduction was not correlated to the reduced RNFL thickness. These results supported the thesis that OAG is a neurodegenerative process, in which involvement is not limited only to neurons located at the retinal level (RGCs) but there is also an impairment of all visual pathway structures responsible for transferring visual information from the eye to the brain.

Demaria et al. studied the relationship between functional connectivity and visual field (VF) loss in primary open-angle glaucoma (POAG) patients compared to healthy controls, evaluated with two resting-state (RS) (f)MRI scans. The authors found no consistent alterations in the global or local functional networks of glaucoma patients, i.e., global brain network communication in glaucoma is preserved. They identified brain areas as being hubs. Among these, the right LIG (Lingual Gyrus) relates consistently with the sensitivity of the binocular integrated VF (BIVF) of participants. The LIG is known for its role in visuospatial processing and topographical recognition, and lesions in this area affect the ability of patients to orient themselves. Frequently, glaucoma patients report difficulties in orientating and moving but this study does not verify cause or consequence of the compromised visual input so future work is needed.

The study of Zhang et al. analyzed retinal nerve fiber layer (RNFL) and retinal ganglion cells (RGCs) alterations in different stages of another neurodegenerative disease, the Amyotrophic Lateral Sclerosis (ALS), and their association with ALS progression parameters. POAG and ALS have several common pathogenic mechanisms, including mitochondrial alterations, axonal transport impairment and high levels of oxidative stress. Moreover, astrogliosis was detected in the subcortical white matter of occipital cortex in ALS and recent studies on ALS supported the involvement of other non-motor systems, including the visual pathways. The RNFL thickness decreases with the disease progression and precedes the RGCs thinning. The significant RNFL thinning in the early stage is related to a faster progression rate and the inverse *U*-shaped curve transformation might be in agreement with early-stage motor neuron axonopathy.

Qiu et al. evaluated the level of sex hormones in female patients with primary open-angle glaucoma (POAG) to determine whether they are associated with the onset and/or progression of POAG. A decreased E2 level was a risk factor for POAG and is associated with VF progression in women with POAG, especially in premenopausal subjects. Additionally, other sex hormones (PROG, LH, FSH, and TESTO) might also play a role in POAG pathogenesis.

Lastly, Arrigo et al. wrote a review about the elements in common between neurocognitive dysfunctions in glaucoma and in neurodegenerative diseases that lead to a cognitive impairment. In this regard, we would like to cite a recent study by Rolle et al. (2019) in which we tested the reading performance, using Radner reading charts, in glaucomatous patients. Indeed our own work fits with the conclusion of Arrigo et al., in particular we found that glaucomatous patients read Radner charts more slowly and inaccurately than controls, and had reading performance that correlated significantly with visual field defects.

In conclusion, the articles published in this Research Topic provide information about the pathogenesis of POAG and the close relationship between glaucoma and brain. They bring further evidences that glaucoma is a neurodegenerative disease that involves cognitive aspects and interferes with patients' quality of life. These studies may provide inputs in new diagnostic strategies, in neuroprotective challenges and also in future therapies, such as brain stimulation (Sabel et al., 2020). Further studies should be focused on the evaluation of the impairment of different visual abilities at earlier stages in order to provide biomarkers of the neurodegeneration, to verify the efficacy of neuroprotective treatments and to identify possible strategies to improve visual abilities, i.e., rehabilitation for these patients.

## Author Contributions

All authors listed have made a substantial, direct, and intellectual contribution to the work and approved it for publication.

## Conflict of Interest

The authors declare that the research was conducted in the absence of any commercial or financial relationships that could be construed as a potential conflict of interest.

## Publisher's Note

All claims expressed in this article are solely those of the authors and do not necessarily represent those of their affiliated organizations, or those of the publisher, the editors and the reviewers. Any product that may be evaluated in this article, or claim that may be made by its manufacturer, is not guaranteed or endorsed by the publisher.
